# A new species of the cleptoparasitic bee genus *Thyreus* from northern Yemen and southwestern Saudi Arabia (Hymenoptera, Apidae)

**DOI:** 10.3897/zookeys.428.7821

**Published:** 2014-07-23

**Authors:** Abdulaziz S. Alqarni, Mohammed A. Hannan, Michael S. Engel

**Affiliations:** 1 Department of Plant Protection, College of Food and Agriculture Sciences, King Saud University, P.O. Box 2460, Riyadh 11451, Kingdom of Saudi Arabia; 2 Current address: 60-125 Cole Road, Guelph, Ontario N1G 4S8, Canada; 3 Division of Entomology, Natural History Museum, and Department of Ecology & Evolutionary Biology, 1501 Crestline Drive – Suite 140, University of Kansas, Lawrence, Kansas 66045, USA

**Keywords:** Apoidea, Anthophila, Melectini, Arabian Peninsula, taxonomy, cleptoparasitism

## Abstract

A new species of cleptoparasitic bee of the genus *Thyreus* Panzer (Apinae: Melectini) is described and figured from northern Yemen and southwestern Saudi Arabia. *Thyreus shebicus* Engel, **sp. n.** is a relatively small species superficially similar to the widespread and polytypic species *T. ramosus* (Lepeletier de Saint Fargeau) and *T. ramosellus* (Cockerell) but more closely allied to various African forms on the basis of the male genitalia. The species is distinguished from its congeners on the basis of coloration pattern, male hind leg structure, and particularly male terminalia.

## Introduction

The cleptoparasitic bee genus *Thyreus* Panzer is one of the more remarkable of Old World lineages. The genus consists of 108 described species (Lieftinck 1962, 1968; Rozen 1969; Eardley 1991; Schwarz 1993; Straka and Engel 2012), and, where known, are cleptoparasitic on species of *Amegilla* Friese (e.g., Bischoff 1927; Popov 1967; Lieftinck 1968 and references therein) and possibly on *Anthophora* Latreille and *Eucera* Scopoli (Stoeckhert 1954; Rozen 1969; Wafa and Mohamed 1970). Species are frequently variable and sometimes even cryptically similar to regionally close taxa, making the group a bane of melittologists. Indeed, although a comprehensive monograph of the group is available (Lieftinck 1962, 1968), it remains a serious challenge to confidently identify several species, particularly those numerous taxa superficially similar to presumably widespread species such as *Thyreus ramosus* (Lepeletier de Saint Fargeau) (e.g., Straka and Engel 2012). Nonetheless, species of *Thyreus* are not infrequently encountered and there is a great potential for detailed biological studies at nesting aggregations of *Amegilla*.

**Figures 1–2.**
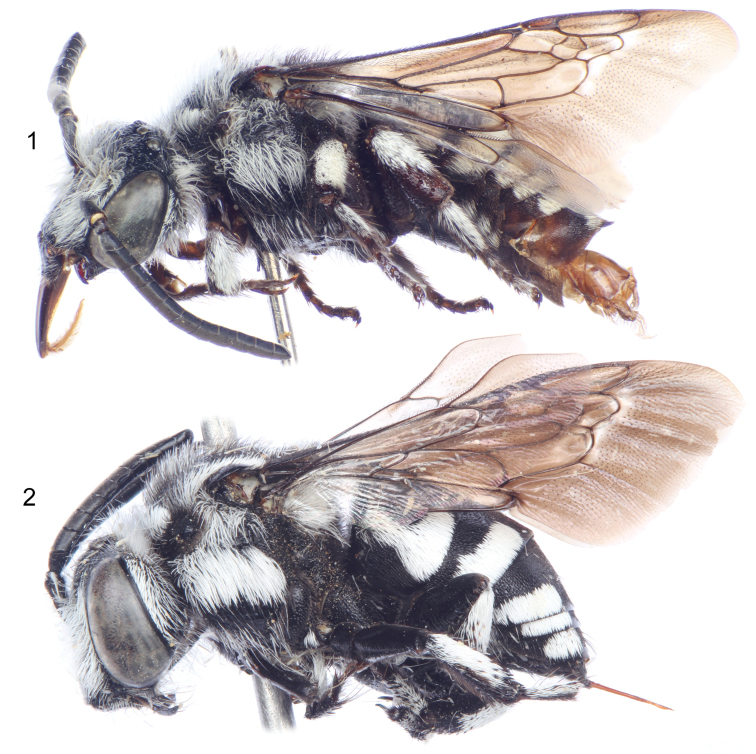
Lateral habitus of *Thyreus shebicus* Engel, sp. n. **1** Male (holotype) **2** Female (paratype).

During ongoing survey work in the Kingdom of Saudi Arabia (e.g., Alqarni et al. 2011, 2012a, 2012b, 2013, 2014a, 2014b, in press; Hannan et al. 2012; Engel et al. 2012, 2013, 2014), a new species of *Thyreus* was discovered in the mountainous areas south from Makkah and this proved conspecific with additional material already known from northwestern Yemen (Figs 1–2). Recognizing the value of descriptive science (Grimaldi and Engel 2007), the species is described herein and compared with its close congeners as an effort to improve the concepts and circumscription of cleptoparasitic bees in the Arabian Peninsula (Engel 2011; Gonzalez et al. 2013), and to encourage melittologists in the region to seek further material along with its host.

## Material and methods

Material is deposited in the King Saud University Museum of Arthropods, Plant Protection Department, College of Food and Agriculture Sciences, King Saud University, Riyadh, Kingdom of Saudi Arabia (KSMA) and Division of Entomology (Snow Entomological Collections), University of Kansas Natural History Museum, Lawrence, Kansas, USA (SEMC). Morphological terminology is based on that of Engel (2001) and Michener (2007), with the addition of those annotations developed by Lieftinck (1962, 1968: *vide etiam* Straka and Engel 2012) for patches of plumose white setae on the mesosoma. As was done by Straka and Engel (2012), we have supplemented the setal patch annontations with their full names inserted in parentheses to ease use while simultaneously maintaining continuity with Lieftinck’s seminal works. The format for the descriptions follows that of Rightmyer and Engel (2003), Straka and Engel (2012), and Engel and Michener (2012). Photomicrographs were prepared with a Canon EOS 7D digital camera attached to an Infinity K-2 long-distance microscope lens and employing a Xenon flash. Measurements were taken with an ocular micrometer on an Olympus SZX12 stereomicroscope.

## Systematics

### Genus *Thyreus* Panzer

#### Thyreus shebicus

Taxon classificationAnimaliaHymenopteraApidae

Engel sp. n.

http://zoobank.org/DEE104F7-E84B-41F8-8355-9CC55C154A23

Figs 1 [Fig F2] [Fig F3] –11

##### Holotype.

♂, N. Yemen, high plateau, 14-4-82 [14 April 1982], I.L. Hamer (SEMC). This is the exact label data from the holotype male and is unfortunately not very precise but likely refers to the mountainous area north of Sana’a and bordering Jazan, Saudi Arabia.

##### Paratypes.

1♀, Saudi Arabia, Asir, Abha, Sawdah [Sodah] (near ropeway), 2670 m, 18°17'37.19"N, 42°21'31.49"E, 22-v-2012 [22 May 2012], M.A. Hannan (KSMA); 1♀, Saudi Arabia: Asir, Abha, Sodah, nr. dam, 2500 m, 18°14'11.64"N, 42°24'49.96"E, 22-v-2012 [22 May 2012], M.S. Engel (SEMC); 1♀, Saudi Arabia, Abha, 6.vi.1972 [6 June 1972], 18.13°42.30°E, A.W. Harvey (SEMC).

##### Diagnosis.

The new species is superficially similar to *Thyreus ramosus* and *Thyreus ramosellus* (Cockerell) but can be distinguished most readily in the form of the male terminalia [*cf.* Figs 5–9 with those in Lieftinck (1968)], particularly in the unique structure of the seventh metasomal sternum and even more extensively with the latter species. In addition, the ventral longitudinal carina of the male metafemur is incomplete (as in *Thyreus ramosellus*, complete in *Thyreus ramosus*) but the apex of the metatibia lacks a comb of dense, long, fine, plumose setae (present in *Thyreus ramosellus*, absent in *Thyreus ramosus*). Females of *Thyreus shebicus* differ from *Thyreus ramosus* and *Thyreus ramosellus* in that *plsa* (anterior posterolateral mesoscutal) does not meet *pls* (posterolateral mesoscutal) and is well differentiated from the latter, and *pls* (posterolateral mesoscutal) is generally smaller in the new species, separated by more than the diameter of an individual *pls* (posterolateral mesoscutal) [distance equal to or frequently less than the diameter of *pls* (posterolateral mesoscutal) in *Thyreus ramosus* and *Thyreus ramosellus*]. The new species may be distinguished from the widespread *Thyreus histrionicus* (Illiger), another superficially similar species, by the more deeply sinuate mesoscutellar posterior margin, the incomplete ventral longitudinal carina of the male metafemur (complete in *Thyreus histrionicus*), the outer surface of the metabasitarsus not concave (concave in *Thyreus histrionicus*), the presence of spots on the male sixth tergum (absent in *Thyreus histrionicus*), and the form of the hidden sterna and genitalia [*cf.* Figs 5–9 to Lieftinck’s (1968) figure 20].

##### Description.

**Figures 3–4. F2:**
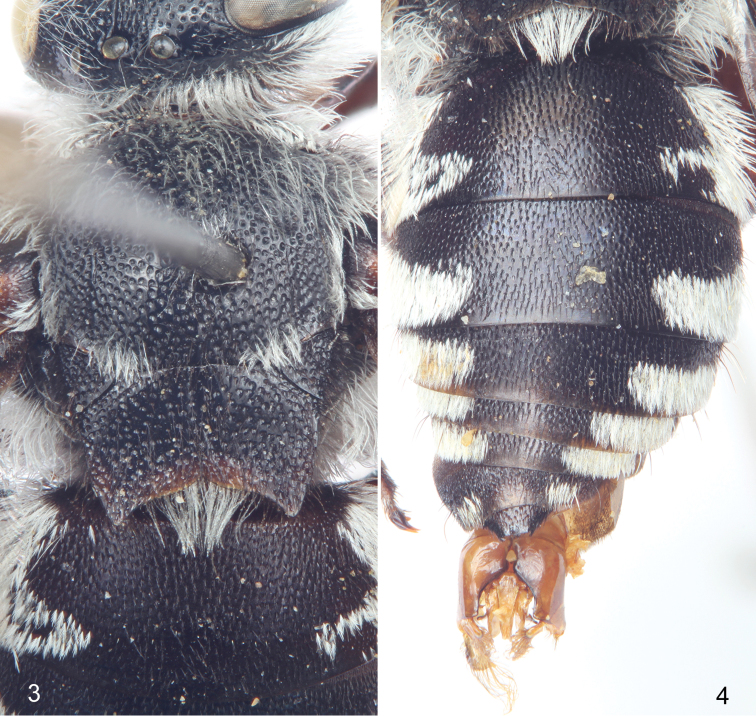
Male (holotype) of *Thyreus shebicus* Engel, sp. n. **3** Mesosomal dorsum **4** Metasomal dorsum.

**Figures 5–9. F3:**
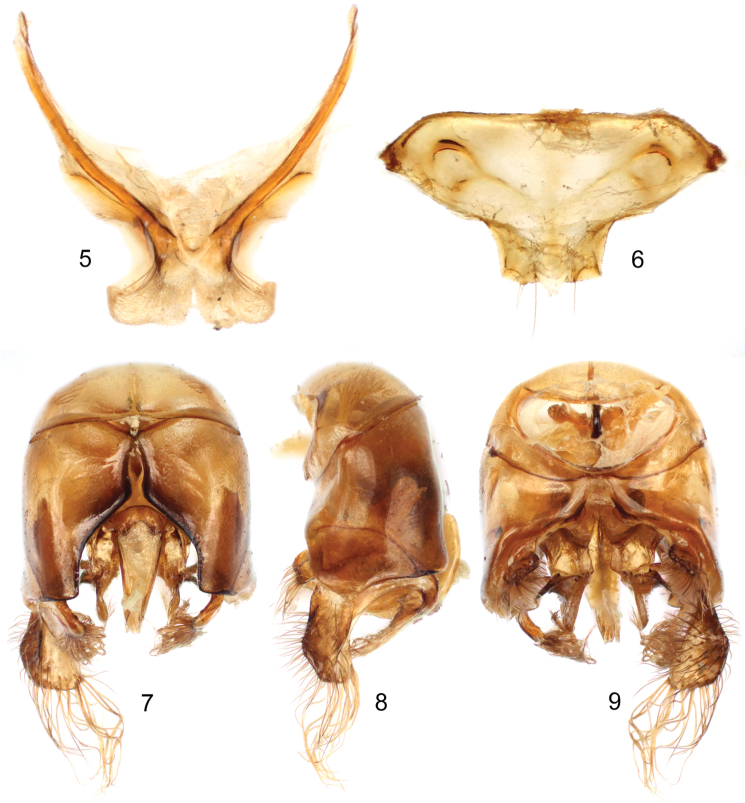
Male terminalia of holotype of *Thyreus shebicus* Engel, sp. n. **5** Seventh metasomal sternum **6** Eighth metasomal sternum **7** Genital capsule, dorsal view **8** Genital capsule, lateral view **9** Genital capsule, ventral view. Note: One gonostylus is missing.

♂: Total body length 10.0 mm; forewing length 7.5 mm. Head wider than long (length 2.3 mm, width 2.9 mm); upper interorbital distance 1.8 mm; lower interorbital distance 1.3 mm. Intertegular distance 2.2 mm; mesoscutellar posterior margin with median emargination, weakly sinuate (Fig. 3), apicolateral angle only weakly produced. Ventral longitudinal carina of metafemur incomplete, carinate only in apical two-thirds, basad carina becomes a defined acarinate angled ridge (in this regard somewhat similar to *Thyreus ramosellus*); inner anterior angle of metatibia not swollen or projecting into prominence or point between metatibial spurs but inner apical border bearing spurs produced gradually outward and posteriorly bordered by apical depressed area with more elongate black setae; apex of metatibia without comb of dense, long, fine, plumose setae; outer surface of metabasitarsus not concave. Apex of seventh metasomal tergum with apicolateral prominences distinct, acutely pointed, length of individual prominence less than one-half of distance between them, truncate margin between prominences straight, without medial emargination or swelling; male terminalia as in Figs 5–9.

Labrum with coarse punctures separated by less than a puncture width except medially and basally separated by a puncture width or slightly less and small, circular, basolateral impunctate areas, integument between punctures smooth, basomedially with shallow, short V-shaped furrow with smaller closer punctures therein; clypeus with small nearly contiguous punctures, integument between smooth; supraclypeal area as on clypeus except punctures sparse medially; lower face as on clypeus except punctures more well defined, becoming progressively larger toward upper frons; punctures become smaller and sparser in ocellocular area, integument between punctures smooth; punctures weaker and shallower on vertex, separated by less than a puncture width immediately posterior to ocelli and bordering preoccipital carina, otherwise rather sparse on vertex; punctures of gena coarse, shallow, and progressively more dense from above to nearly contiguous by midlength; postgena finely imbricate and impunctate. Pronotum with coarse, shallow punctures separated by a puncture width or less, integument between smooth to faintly imbricate; mesoscutum with well-defined, coarse, contiguous punctures laterally (Fig. 3), punctures slightly more widely spaced medially such that punctures separated by about 0.25–0.5 times a puncture width, integument between punctures smooth; axilla with punctures contiguous; mesoscutellum with punctures as on mesoscutum except separated by 0.25–0.75 times a puncture width, more closely spaced laterally; pleura with coarse, nearly contiguous punctures, integument between punctures (where evident) finely and faintly imbricate, punctures of mesopleuron ventrally becoming more elongate and widely spaced, punctures of preëpisternal area and metapleuron smaller than those of mesopleuron and contiguous; hypoepimeral area large, coarse, nearly contiguous punctures; propodeal lateral and posterior surfaces with coarse, shallow, ill-defined, nearly contiguous punctures. Metasoma with small punctures separated by a puncture width or more often less (Fig. 4), punctures more coarse, larger, and somewhat more poorly defined on discs of more apical terga, integument between faintly and finely imbricate, apical margins narrowly impunctate and finely imbricate; sterna with similar punctation except those on discs of more basal sterna more widely spaced and becoming more poorly defined on more apical sterna.

Integument black except dark brown on labrum, mouthparts, legs, and apically on mesoscutellum, seventh metasomal tergum, and on apical sterna. Wing membranes hyaline and slightly infumate except with whitish along apical border of 2rs-m and 2m-cu (Figs 1–2), veins dark brown to black.

**Figures 10–11.**
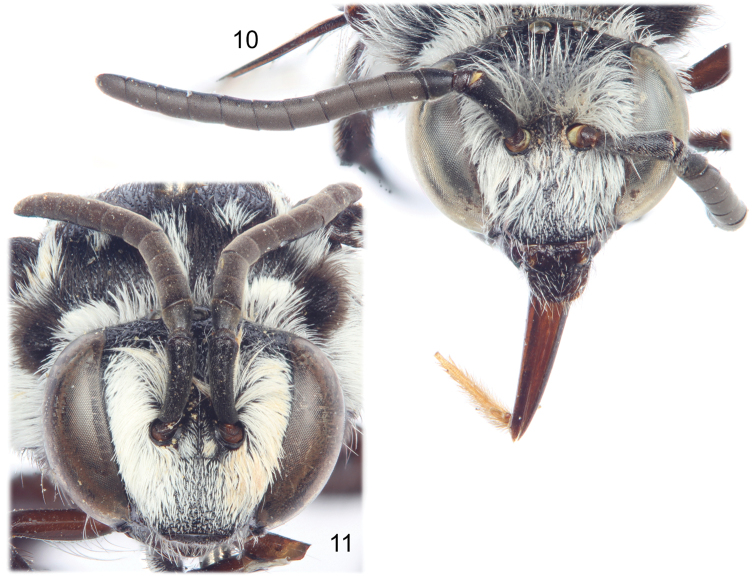
Faces of *Thyreus shebicus* Engel, sp. n. **10** Male (holotype) **11** Female (paratype).

Pubescence generally fuscous to black over entire body except in the presence of long plumose white setae over most of face (Fig. 10), posterior on vertex, ventral margin of mandible, entire gena, postgena, outer surface of protibia and probasitarsus (although white setae appressed and short on this surface), outer surface of mesotibia and mesotarsus (appressed on these surfaces), apical ventro-posterior border of mesofemur, outer posterior angles of meso- and metacoxae, outer surface of metatibia and metarasus (appressed on these surfaces), and on mesosoma (using the annotation system of Lieftinck 1962, 1968) as follows: *deps* (dorsal mesepisternal) and *lpn* (lateral pronotal) present; *als* (anterolateral mesoscutal) present but diffuse and faint; *ms* (median mesoscutal) present but diffuse and faint; *mls* (mediolateral mesoscutal) present albeit very diffuse; *plsa* (anterior posterolateral mesoscutal) present along border with tegula, not meeting *pls* (posterolateral mesoscutal) posteriorly; *t* (tegular) present and prominent posteriorly on tegula; *pls* (posterolateral mesoscutal) present, not extending laterally to meet *plsa* (anterior posterolateral mesoscutal); *ps* (parascutellar) and *s* (mesoscutellar) absent; *deps* (dorsal mesepisternal), *hypm* (hypoepimeral area), and *lp* (lateral propodeal) present, *veps* (ventral mesepisternal) present albeit diffuse (Figs 1, 3) (much of these white patches are diffuse in the male and partially rubbed off as preserved, most patches more well defined in female). Mesoscutellum with patch of long, plumose, white setae extending posteriorly from undersurface of mesoscutellum medially, patch wide but not reaching to apicolateral corners. Metasomal terga with prominent patches of appressed, plumose white setae as follows: first metasomal tergum with large, L-shaped patches laterally; second metasomal tergum with lateral patch L-shaped although transverse section more well developed; third through sixth metasomal terga with transverse lateral patches, those of sixth tergum more rounded (Fig. 4).

♀: As described for male except in usual gender differences and as follows: Total body length 8.1–9.9 mm; forewing length 7.1–8.1 mm. Head wider than long (length 2.3–2.6 mm, width 2.9–3.4 mm); upper interorbital distance 1.8–1.9 mm; lower interorbital distance 1.3–1.5 mm. Intertegular distance 2.3–2.7 mm; mesoscutellar posterior margin as in male but sometimes sinuate margin weaker and posterior angles more prominent. Pygidial plate relatively narrow, lateral margins largely straight and converging apically, apex broad and truncate, surface imbricate and impunctate, apically with weak medial carina.

Mesoscutal punctures slightly more spaced than in male; metasomal terga with punctures generally separated by less than a puncture width, apical margins narrowly impunctate and imbricate except apical margin of fifth tergum broadly impunctate and imbricate, covering approximately apical half.

Integument and pubescence as in male except reddish brown on pygidial plate; white mesoscutal setal patches generally more well defined and not as diffuse as in male; second through fifth metasomal terga with transverse lateral patches (Fig. 2).

##### Etymology.

The specific epithet refers to the ancient kingdom of Sheba, realm of the Queen of Sheba and the people of Tubba’, and likely consanguineous with the Sabaeans who occupied several of those areas in the southwest of the Arabian Peninsula in which the species here has been taken.

## Discussion

At first glance the new species could easily be confused with the widespread *Thyreus ramosus* and, despite some of the difficulties with Lieftinck’s (1968) key, will generally run to that taxon if the ventral longitudinal carina of the male metafemur is ignored. Otherwise, based on this feature, *Thyreus shebicus* would run to *Thyreus ramosellus*. Nonetheless, there are profound differences in the structure of the male terminalia between *Thyreus shebicus* and *Thyreus ramosus* and *Thyreus ramosellus* [*cf.* Figs 5–9 with Lieftinck’s (1968) figures 32–33, 37–38]. Moreover, the form of the mesoscutellum and male seventh metasomal tergum in *Thyreus shebicus* do not match those of either of the aforementioned species. In fact, many forms identifiable as *Thyreus ramosus* or *Thyreus ramosellus* sensu Lieftinck (1968) may represent distinct, perhaps even cryptic species, and there is a general need for a revised circumscription of the taxa within this complex and perhaps across the entire genus, as is necessary for many bee genera where concepts of species have not been tested in a generation or more (Gonzalez et al. 2013). Among African species there are those that closer approximate *Thyreus shebicus* in the structure of the hidden sterna, such as *Thyreus abyssinicus* (Radoszkowski) and *Thyreus brachyaspis* (Cockerell), but the terminalia and genital capsules among all three remain specifically distinct and the patterns of coloration and other structural features (e.g., form of the seventh metasomal tergum) are further different (*vide* Eardley 1991). Nonetheless, this general similarity between African and western Arabian taxa is a general pattern observed across several insect groups and also matches with the geological history of the region (e.g., El-Hawagry et al. 2013; Engel et al. 2014). Notwithstanding the apparent patterns mentioned, a comprehensive phylogeny of *Thyreus* is necessary to tests these hypotheses and reveal the complicated biogeographic history of the clade.

Admittedly, *Thyreus* is not only a large and diverse group, encompassing a vast distribution throughout the Old World, but the distinctions between natural units have been challenging to discern. Lieftinck’s herculean effort was certainly a massive leap forward and some of his hypotheses for widespread and rather polytypic species require further testing, ideally in combination with genetic data. In addition to the aforementioned *Thyreus ramosus* and *Thyreus ramosellus*, another such suspicious species is *Thyreus nitidulus* (Fabricius) (*vide* Lieftinck 1959). Such work is not only pertinent for the proper characterization of species within the genus, but will permit eventually a comprehensive phylogenetic and biogeographic study across the clade, and, should sufficiently-large numbers of host nests be discovered to provide a ready supply of developmental stages of species of *Thyreus*, then perhaps the unique mesoscutellar shield of *Thyreus* could be explored from an evolutionary developmental perspective. Differential gene expression comparisons between developing *Thyreomelecta* Rightmyer and Engel and basal members of *Thyreus* would be revealing as to the underlying genetic architecture of morphogenesis in these bees.

Several suitable species of *Amegilla* were found flying in the same locality as *Thyreus shebicus*. Any or multiples of these may serve as the host for this species and only through discovery of the nests of the *Amegilla* will it be possible to ascertain the specific association of the cleptoparasite. Continued intensive sampling and hunting for nests of bees in southwestern Saudi Arabia and particularly in the area around Abha is needed. Presently documented species of *Thyreus* from the Arabian Peninsula include the regionally widespread *Thyreus elegans* (Morawitz) and *Thyreus ramosus*, *Thyreus ramosellus* in the northern parts, *Thyreus parthenope* Lieftinck in largely the west and south of the peninsula (western Saudi Arabia and Yemen), and *Thyreus fallibilis* (Kohl) in the southwest [originally described from ‘South Arabia’, a former name for the Republic of Yemen and some parts of Jazan, Asir, and Najran (*N.B.*: ‘S. Arabia’ on many pre-1968 labels refers to ‘South Arabia’, of the Federation of South Arabia or the Protectorate of South Arabia, and not Saudi Arabia)].

## Supplementary Material

XML Treatment for Thyreus shebicus
